# Volunteering among Chinese College Students during the COVID-19 Pandemic

**DOI:** 10.3390/ijerph19095154

**Published:** 2022-04-23

**Authors:** Yun Geng, Shannon P. Cheung, Chien-Chung Huang, Jinyu Liao

**Affiliations:** 1 School of Government, Central University of Finance and Economics, Beijing 100081, China; yungeng@cufe.edu.cn (Y.G.); liaojinyu0417@163.com (J.L.); 2 School of Social Work, Rutgers University, New Brunswick, NJ 08901, USA; scheung@ssw.rutgers.edu

**Keywords:** volunteering, college, altruism, China, public interest, private gains, well-being

## Abstract

Volunteering has been found to be not only beneficial to the well-being of recipients but also to the volunteers themselves, particularly from the life course perspective. Although previous studies have identified key factors of volunteering motivation, the literature is less focused on the interplay of public interest and private gains in volunteering motivation. This study used 1871 college students across China to examine how the interplay between public interest and private gains affects general and Coronavirus Disease 2019 (COVID-19)-specific volunteering during the pandemic. The results show that the interplay of these two factors constitutes a dynamic process, depending on the volunteering and time-specific context. Overall, undergraduate students with greater concern for public interest and less preference in private gains had the highest rate of overall volunteering, followed by students with high concern for both public interest and private gains. It is crucial to take both public interest and private gains into account when discussing volunteering opportunities among Chinese college students, which may increase the well-being of students in the long run.

## 1. Introduction

### 1.1. Volunteering among Chinese College Students during the COVID-19 Pandemic

Volunteering is an activity in which an individual or a group of individuals freely give their time and labor to benefit another person, group, or cause [1]. Volunteering has been found to be not only beneficial to recipients but also to the volunteers themselves [1,2,3]. Positive effects experienced by volunteers include greater life satisfaction and occupational achievement. Volunteering is also linked to reduced problem behaviors such as drug abuse among adolescents and youths [4,5,6,7,8], as well as the general population [9,10,11].

About 80% of college students in China volunteer, which is comparable to rates of students in USA and Canada but higher than the volunteering rate of students in Japan [12,13]. The high rate of volunteering in Chinese college students is partly due to the strong state-led emphasis on community service [13,14,15] and partly due to the Confucian value of benevolence and collectivistic orientation in Chinese culture [16,17].

Many studies have examined antecedents of volunteering from different perspectives, including psychological, sociological, and economic viewpoints [12,18,19,20,21,22,23,24,25,26,27,28]. Psychological perspectives focus on intrapsychic phenomena such as personality traits and motivation. Sociological perspectives emphasize socio-demographic characteristics, such as education, religion, social class, and environment, while economic perspectives discuss cost-benefit analyses in the context of volunteering. Studies have found that personality traits such as altruistic motivation and empathy were positively related to volunteering [25,29,30]. Individuals with high educational attainment and social capital [1,27,31] and those surrounded by a positive school culture and environment [32,33] also tend to be more involved in volunteering efforts than their counterparts. Finally, intrinsic and extrinsic benefits and rewards, including prestige and training, are positively associated with volunteering [19,28,34,35,36,37,38].

The outbreak of a novel coronavirus disease (COVID-19) led to its rapid spread throughout China and the rest of the world. The outbreak caused serious public health threats around the world [39,40,41]. At the time of writing this paper, 2 April 2022, Johns Hopkins Coronavirus Resource Center (CRC) estimates that there are more than 490 million cases of COVID-19 infections and more than 6.1 million people who have died of COVID-19-related causes [42]. Studies have shown that, though the morbidity and mortality rates of COVID-19 were not high in China [43,44,45], the strict home-quarantined policies have had negative effects on mental health outcomes. These include increased stress, anxiety, and depressive symptoms [46,47,48,49]. Recent studies have also studied the motivational factors of volunteering during the COVID-19 pandemic for medical students [50,51,52,53,54,55] and community residents [56,57], but less so for undergraduate students. The findings suggest that volunteering during the pandemic is related to not only altruism but also professional identity, operational, and intrinsic and extrinsic motivational factors [52,53,54,55,56,57].

The college years have been found to be a particularly important time as this period is characterized by increasing independence and responsibility [58,59]. From the life course perspective, volunteering in college may have long-term and positive effects on later achievement and well-being [4,6,7,8]. The study of factors that increase volunteering are important to shed light on the human capacity to thrive in the face of challenging life circumstances, including those that may occur during a critical transitory period between adolescence and adulthood [58]. Thus, it is important to understand the antecedents of volunteering in college as well as to assess whether the COVID-19 pandemic affects volunteering during college.

The literature has shown that individuals with altruistic motivation and empathy are more likely to volunteer out of concern for public interest [18]. The literature has also found that those who seek out high intrinsic and extrinsic benefits and rewards are likely to participate in volunteering out of interest in private gains [28,35]. As recent studies point out volunteering motivations may stem from both public and private ones [51,52,53,54,55,56,57], it is important to know how these two separate constructs interact with one another to influence volunteering motivations. To test for the interplay of public interest and private gains in volunteering motivation, we develop a vignette to examine how these two constructs, concern for public interest and investment in private gains, affect Chinese undergraduate students’ engagement in volunteering during the COVID-19 pandemic.

### 1.2. Volunteer Motivation: Public Interest vs. Private Gains

**Figure 1:**
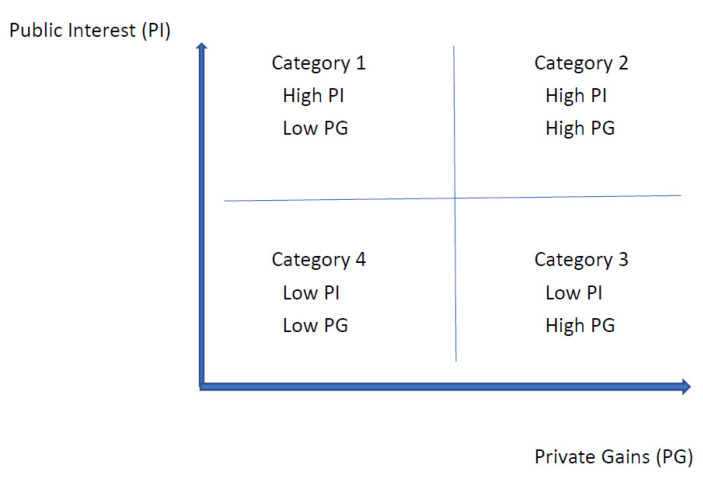
Interplay of Public Interest and Private Gains.

We contend that motivation to volunteer is determined by individuals making decisions based on both public interest and private gains. Public interest refers to the welfare or well-being of the public, and private gains are the intrinsic and extrinsic benefits and rewards for individuals [28,35,60]. Public interests and private gains are interdependent in many public policies and private behaviors [60,61]. In this study, we argue that a person decides whether to volunteer for some event based on the public and private gains and losses that this event may pose. Using public interest and private gains as two axes, we categorize each type of motivation into four categories, as shown in Figure 1.

The first category represents high public interest and low private gains. A person with this classification of motivation would have a strong sense of concern for public well-being and a low investment in private gains when electing to participate in a volunteering activity. Individuals in this category participate in volunteering simply to impart benefits to others and less so for the personal gains. Thus, as long as the volunteer activity appears to benefit public interests, the likelihood and extent of this person’s volunteering will be greater, even if the activity itself does not align with their personal interests. 

The second category indicates high concern for public interest and high investment in private gains; a person in this category has a strong sense of concern for public well-being as well as their own personal gain when participating in a volunteering activity. People in this category volunteer because the activity benefits the public and themselves; in other words, the volunteering is conceptualized as a rewarding experience [28,62]. If the activity does not offer high benefits for the public interest or individual, the likelihood and the extent of volunteering would be low for the activity. 

The third category is low concern for public interest and high investment in private gains. People in this category participate in volunteering mostly for their own personal gain and less so out of concern for the public. As long as the volunteer activity offers benefits for the individual, they will be more likely to engage in the activity, even if the activity offers minimal benefits for others. The fourth and final category is low concern for public interest and investment in private gains. Those in this category would not volunteer for neither public interest nor personal gain. Thus, if the volunteer activity offers minimal benefits in both realms, the likelihood and the extent of the individual’s volunteering would also be low for the activity.

In short, we propose that a person chooses to volunteer based on the interplay of public interests and private gains as public interests and private gains cannot be fully understood if they are conceived independently [60,61]. 

## 2. Data and Method

### 2.1. Data and Sample

The data for this research were from an online survey of college students in China. The inclusion criterion was that participants had to be either a junior or senior social science student. The sample was limited to junior and senior students so as to assess the extent of volunteering of students who had experienced at least one year of college prior to the pandemic. The sampling procedure was designed to have reach a large, geographically diverse sample that would be sufficient to conduct multivariate analysis. Twelve leading universities were selected across the northern, eastern, southern, western, and central regions of China. Once universities were selected, we reached out to departments of social science, yielding a sampling frame of 2229 students. We invited students to participate based on an incentive of 10 RMB for participation (2 USD) in late September 2020. Reminders for invited students were sent three and seven days later. Prior to beginning the survey, students were informed of their voluntary participation and their ability to discontinue the survey at any time. They were also informed that their survey would be kept anonymous, with no personal information collected, and would have no bearing on their academic standing. Students, on average, took 15 min to complete the survey. This research protocol was approved by the research review committee at one of the co-authors’ university. In total, 1881 students participated in the online survey by early October 2020. Ten students had incomplete answers and were excluded from the final analysis. Our final analytic sample contained 1871 students. The response rate of the survey was 83.9%.

### 2.2. Measures

The dependent variable, volunteering, was measured by whether the student had participated in any volunteer activity in the last year. If students answered yes, they were also asked about frequency (how many times) and time spent volunteering in hours. We also asked whether students volunteered for COVID-19 relief efforts. For those who answered yes, we also asked about frequency and time spent volunteering in the last year.

The key independent variable is the interplay between concern for public interest and investment in private gains. We created a vignette to measure the interplay by the question, “A nonprofit organization launches a new project that aims to improve the well-being of a disadvantaged group, and volunteers are required to allocate 10 h per month for a period of six months (for a total of 60 h). Would you participate?” The potential answers included:

1. No matter what the project is, I will participate as long as the project helps others;
2. I will participate only if the project fits with my interests;
3. I will participate only if participation counts as course credit; and
4. No matter what project is, I do not have any interests in participating.

The first answer option represents high public interests and low private gains, while the second indicates high public interest and private gains, as the hypothetical project itself is being launched by a nonprofit organization for the well-being of a disadvantaged group. The third answer represents low public interest and high private gains. Finally, the fourth answer indicates low public interest and private gains.

We controlled for COVID-19 infection among respondents’ family and friends by asking whether the subjects’ family members and friends had been infected with or died from COVID-19. We also controlled for students’ socioeconomic characteristics in this study. These characteristics include age, gender (0 = male; 1 = female); ethnicity (1 = Han; 0 = other); household registration (rural, city with prior, and city); parents’ marital status (married, separated, divorced, and widowed); parents’ highest educational attainment (elementary school or below, middle school, high school, and some college or above); number of family members; and annual family income and welfare status (0 = no; 1 = yes) in the last year. Finally, as previous studies have shown that school environment and local culture make differences in volunteering engagement [32,33], we took college characteristics into account by controlling for specific college influence, or college-fixed effect.

### 2.3. Analytical Strategy

We first conducted descriptive analysis to examine the distribution of main variables. Regression analysis was then performed to assess the net effects of key independent variables on the dependent variable while controlling for socioeconomic characteristics of students and specific college effect (i.e., college-fixed effect). The framework underlying this study posits that the extent of volunteering among college students is determined by the interplay of public interest and private gains, COVID-19 infection in family and friends, and socioeconomic and university characteristics of the students. The three outcomes of volunteering include whether a student engaged in any volunteering; volunteering frequency, and total number of hours spent volunteering. Engagement was a dichotomous variable, with 1 representing that a student had volunteered at least once, and 0 representing that they did not volunteer at all. Logit regression was used to estimate the net effects of the explanatory variables on the volunteering engagement. As for volunteering frequency and time spent volunteering, ordinary least squares (OLS) regression was used for the analyses. The natural logs of volunteering frequency and time spent volunteering were used to account for high ranges and skewness of student reports. For students with no engagement in volunteering activity, we added 0.1 to both frequency and time spent before the log transformation. 

## 3. Results

### 3.1. Descriptive Statistics

Table 1 presents the descriptive statistics for the main variables. About three out of four students (74.93%) engaged in volunteering in the last year. On average, students volunteered 3.29 times and spent 24.73 h volunteering. About one in four students (25.12%) had volunteered for COVID-19 aid. These students who volunteered 0.66 times and for 4.14 h. Students volunteered during the COVID-19 pandemic less than previously across all three dimensions. A majority of students (62.53%) chose the second answer when indicating how the interplay of public interest and private gains affected their decision to volunteer. That is, approximately 63% of students chose to volunteer when the activity had benefits for both the public interest and themselves. This was followed by the first category, high public interest and low private gains (27.74%); third category, low public interest and high private gains (5.99%); and fourth category, low public interest and private gains (3.74%). 

Although the news of COVID-19 constantly permeates daily life, a majority of students did not have any family members or friends who were infected with or died from COVID-19 (99.14%). Only nine (0.48%) and seven (0.37%) students reported they had family members and friends infected or died from COVID-19, respectively. Given the small percentages for each category, we combined both into one, “infected,” in our analyses.

### 3.2. Multivariate Analyses

#### 3.2.1. Motivating Factors for Volunteering

Table 2 presents the logit-regression estimates of volunteering. Four models are presented. The first two models regressed overall volunteering activity onto our independent variables. The first model includes COVID-19 infection and socioeconomic characteristics of the students, while the interplay between public interest and private gains was added into Model 2. The last two models are exactly same as the first two, except that the dependent variable was engagement in COVID-19 volunteering.

Gender and grade had significant effects on volunteering in Model 1. Female students had greater odds (1.30) of volunteering than male students. Junior students also had greater odds (2.52) of volunteering than senior students. Since the latter are more likely to be in the job market searching for employment, this may have limited their availability to volunteer. Age and welfare status showed marginal significant effects on volunteering. Older students and those whose family received welfare were more likely to volunteer. After we added the interplay between public interest and private gains to Model 2, socioeconomic characteristics, such as gender, age, and welfare status, were no longer significant. In this model, only the interplay between public interest and private gains and grades significantly affected students’ volunteering engagement. Compared to students in the fourth category (low public interest and private gains), the odds of those in the first category (high public interest and low private gains) was 5.01. Likewise, the odds of students in the second (high public interest and private gains) and third categories (low public interest and high private gains) of volunteering were 3.45 and 2.98, respectively. Parental education also had positive and marginal significant effects on volunteering.

Turning to the results of the COVID-19 volunteering models, Model 3 indicated that gender, age, grade, parents’ highest education attainment, and welfare status had effects on COVID-19 volunteering. However, unlike in Model 1, in Model 3, female students were less likely to engage in COVID-19 volunteering than their male counterparts. Older students, those whose parents had received a high school education, those in their junior year, and those whose families had received welfare were all more likely to participate in COVID-19 volunteering than their counterparts. 

The interplay between public interest and private gains had a significant effect on COVID-19 volunteering in Model 4. Compared to the fourth category, students in the first category had significantly greater odds of participating in COVID-19 volunteering (odds = 2.30). In short, the results in Table 3 suggest that the interplay between public interest and private gains was an important factor for overall volunteering, but its effect on volunteering during the COVID-19 pandemic was relatively smaller. The motivating factors for COVID-19 volunteering were more related to socioeconomic characteristics of the students, such as gender, age, and grade. 

#### 3.2.2. Motivating Factors for Frequency of Volunteering

Table 3 lists the standardized estimates of the natural log of volunteering frequency, estimated by OLS regression. For simplicity, the two full models in Table 2 are presented in Table 3. The results of volunteering frequency in the last year (Model 1) show that the interplay between public interest and private gains, grade, parents’ highest educational attainment, and welfare status had significant effects on students’ volunteering frequency in the last year. Compared to those with low public interest and private gains, the other three categories were significantly positively associated with volunteering frequency, with standardized coefficients of 0.34, 0.27, and 0.11 for the first, second, and third categories, respectively. Compared to senior students, junior students volunteered more frequently in the last year. Students of parents with a high school education and students whose families received welfare reported greater volunteering frequency than their counterparts.

The results in Model 2 of Table 3 show that the interplay between public interest and private gains had effects on COVID-19 volunteering frequency. Compared to those with low public interest and private gains, the first category had significantly positive effects on the frequency of COVID-19 volunteering, with standardized coefficients of 0.15. Female students volunteered less frequently during the pandemic than male students. Age increased COVID-19 volunteering frequency. Compared to senior students, junior students had had higher COVID-19 volunteering frequency in the last year. Students whose parents had a high school education and students whose families had received welfare reported higher COVID-19 volunteering frequency than their counterparts.

#### 3.2.3. Motivating Factors for Hours of Volunteering

Table 4 lists the standardized estimates of the natural log of hours spent volunteering in the last year, estimated by OLS regression. Similar to Table 3, two models are presented in Table 4. The results of Model 1 indicate that the interplay between public interest and private gains made a difference in the total hours spent volunteering in the last year. Compared to those with low public interest and private gains, all other categories had significant positive effects on the hours spent volunteering, with standardized coefficients of 0.30, 0.27, and 0.11 for the first, second, and third categories, respectively. Compared to senior students, junior students spent more time volunteering in the last year. Students whose parents had completed junior high school and high school reported spending more time volunteering than their counterparts. 

The results of Model 2 in Table 4 show that, compared to those with low public interest and private gains, the first category of interplay had significantly positive effects on hours spent on COVID-19 volunteering, with a standardized coefficient of 0.14. Female students spent fewer hours volunteering than male students. Age increased time spent on COVID-19 volunteering. Compared to senior students, junior students spent more time volunteering during the COVID-19 pandemic. Students whose parents completed high school reported spending more time volunteering during COVID-19 than did their counterparts.

## 4. Discussion

### 4.1. Volunteering in Chinese Undergraduate Students

The literature has shown that the antecedents of volunteering include altruistic motivation for improving public welfare as well as rational decisions based on private benefits and gains [5,25,28,34]. Recent studies on volunteering during the COVID-19 pandemic suggest that both public interest and private gains influence the decision to volunteer during the pandemic [51,52,57]. Less is known about the interplay of these two factors of volunteering engagement among undergraduate students, especially in China. This study used data collected from 1871 college students across China to examine how this interplay affects both general volunteering and COVID-19-specific volunteering during the COVID-19 pandemic.

The results show that about three out of four Chinese college students volunteered last year at an average frequency of about 3.3 times per year. On average, students reported volunteering about 25 h during the last year. By contrast, only one in four students participated in COVID-19 volunteering, volunteering an average of 0.7 times and about 4 h. The results indicate that a majority of Chinese college students had volunteered during the past year, but their volunteering activities dropped substantially during the COVID-19 pandemic; this is different from the youth volunteering after Wenchuan earthquake in 2008 and may be a result of heightened health risks during the pandemic, which could have deterred students from volunteering [56,63,64].

The percentage of students in our sample who volunteered is comparatively lower than that of Chinese students who volunteered in 2010 (around 84%) but similar to those percentages of college students based in USA, Canada, Belgium, and Finland, which were all between 70 and 80% [12,13]. Relative to other Asian countries, this sample had a higher than volunteering rate than students in Japan (39.1%), similar to Korean students (73.0%), and lower than Indian students (86.2%) [12]. The varied percentages across countries raise an important research question: do volunteering motivations differ in relation to cultural and political contexts? Indeed, for example, recent studies have shown that religion may play an important role in volunteering motivations and behaviors in European countries [51,54,55]. The Confucian value of benevolence, along with strong state-led emphasis on community service, may play a role in volunteering within Chinese society [13,14,15,16,17]. Future studies are warranted to examine cultural and political factors in volunteering motivations and behaviors across different country contexts.

### 4.2. Interplay of Public Interest and Private Gains in Volunteering

A majority of Chinese students take both public interest and private gains into consideration when choosing whether to engage in a volunteering opportunity. Indeed, 62.53% of students report that they participate in volunteering only when it aligns with their investment in personal gains and with the concern of public interest. About one quarter of students (27.74%) valued public interest, largely neglecting private gains, as the sole reason to volunteer. A small proportion of students (5.99%) stated that they would volunteer only when participation counted for course credit; these students did not consider public interest as a factor in their decision-making. Lastly, an even smaller proportion of students (3.7%) did not intend to volunteer at all, regardless of the project and its possible benefits for the public or individual. Overall, about 90% of Chinese college students considered how the volunteering activity would impact public interest when considering participation; a majority also considered take private gains when making this decision.

The interplay of concern for public interest and investment in private gains has significant effects on overall volunteering (participation, frequency, and time) during the COVID-19 pandemic. A consistent pattern between the interplay of these two factors and volunteering was identified within all three dimensions of volunteering. Students with greater concern for public interest and low investment in their private gains (the first category) tended to volunteer the most; they also volunteered more frequently and had the most time spent volunteering than others. These results were followed by students with high public interest and private gains (the second category), then students with low public interest and high private gains (three category). The fact that the estimated coefficients for students in the first category were higher than for those in the second category indicates that genuine concern for the public promotes volunteering engagement the most. In addition, the estimated coefficients for students in the second category were higher than for those in the third category, showing that a balance of concern for public interest and investment in private gains is a better motivator than simply an investment in private gains. Finally, the estimated coefficients were all significant for the third category compared to the fourth category indicate that underscoring the personal benefits of volunteering would motivate students with low public interests and private gains.

### 4.3. Interplay of Public Interest and Private Gains in COVID-19-Specific Volunteering

However, when we examined COVID-19-specific volunteering, these results differed. Only students in the first category were more likely to participate in COVID-19 volunteering when compared to others. These students also volunteered more frequently and had spent more time on COVID-19 volunteering than others. Volunteering activity of students in the second and third categories did not significantly differ from that of students in the fourth category. The findings show that concern for public interest promoted COVID-19 volunteering the most, while other students hesitated to get involved, possibly due to perceived high health risks when considering the highly contagious nature of COVID-19. The findings are consistent with studies on the mental health outcomes of college students during COVID-19 pandemic, which showed that students faced high likelihood of stress, anxiety, and depression [46,47,48,49]. As a result, volunteer motivation is less likely to affect their behaviors when they must face their own personal challenges and are also in need of support [65,66,67]. Further research on COVID-19 specific volunteering is warranted.

### 4.4. Policy and Practical Implications

These findings may provide policymakers the tools to encourage volunteering within student populations. In the last twenty years, high schools and colleges have started to require volunteering as a part of their curriculum. Some have also established for-credit service-learning courses. Studies have found that the volunteering and service-learning experience, regardless of whether these opportunities are voluntary or required, are strong predictors of volunteering and well-being in adulthood [68,69,70,71]. If the above curricula and programs can encourage students who are classified in the fourth category to move into the third, second, or first category, this may increase their volunteering involvement and well-being in the long run.

Although we found that the first three categories of the interplay between public and private interests encouraged overall volunteering in this study, they showed quite different distributions within the sample. Students who valued both public interest and private gains were the largest subgroup, followed by those who valued public interest more and private gains less. This subgroup also showed the most consistent volunteering both overall and during the COVID-19 pandemic. The third subgroup, which valued public interest less but private gains more, were still somewhat involved in volunteering and may serve as a channel for the last group to engage in volunteering. Policy and practice such as for-credit service-learning curricula can move those students in the fourth group into the third. Further research is warranted to study the dynamics and movements across these four categories over time.

In addition, given the low percentage of COVID-19 specific volunteering found in this study, along with challenges found in volunteering during the pandemic in previous studies [50,51,52,56], policies need to be developed to have a system that prepares student volunteers for the next health crisis. Such a system can include approaches to promoting altruistic motivations among students, enhancing students’ social skills and knowledge of health safety, and supporting the transitional phase from students to professional volunteers. This last approach requires strategizing local logistics, establishing a volunteer management team, and providing intrinsic and extrinsic incentives and psychological support to student volunteers during crisis [50,51,52,53,54,55,56].

### 4.5. Limitations

The results of this study must be contextualized within its limitations. First, our analyses were based on a cross-sectional dataset, which can only approximate an associative relationship, rather than a causal one, between public and private factors and volunteering. In particular, voluntary motivation was measured by a hypothesized question. As a result, we were unable to determine the chronological order of the variables. A future study that uses longitudinal design to examine the chronological order of motivation and behavioral outcomes is warranted. Second, there may be other unobserved variables that affect volunteering but were not included in the study. The absence of these unobserved variables may have effects on the estimates reported in this study.

Third, the generalizability of these findings to the larger college student population is limited since our data were from social science students only. Although we aimed to have a large sample size of students from geographically diverse colleges to increase the confidence of our results, the extent to which our findings may represent all Chinese college students is unknown. Further research may seek to recruit students from more varied disciplines.

Fourth, although we found that proximity to COVID-19 infection was not a statistically significant predictor of volunteering, this may be a result of the fact that few students reported positively for this variable. Indeed, less than 1% of the sample reported that their family members and/or friends had been infected with or died from COVID-19. Future research using samples from different cities or countries may produce different results. Finally, the vignette used to measure the interplay between concern for public interest and private gains was based on single question and needs to be further tested for reliability and validity. Alternatively, researchers can develop a scale to measure the interplay. Despite the limitations mentioned above, the present study contributes to the knowledge of decision making when it comes to Chinese undergraduate student engagement with volunteering opportunities during the pandemic.

## 5. Conclusions

The findings in this study suggest that the interplay of these two factors, concern for public interest and investment in private gains, constitutes a dynamic process, depending on the volunteering context during the pandemic. Students value both public interest and private gain in participating general volunteering during the pandemic; however, when the volunteering is specific to COVID-19, which might be associated with increased health risks, only public interest value promotes COVID-19 specific volunteering during the pandemic. This calls for more research on volunteering to consider individuals’ concern for public interest and investment in private gains in Chinese college students and beyond.

## Figures and Tables

**Table 1:** Descriptive Statistics of Key Variables. Note: *n* = 1871.

	Mean (S.D.)
Gender [%]	
Female	66.97
Male	33.03
Age	20.62 (0.96)
Household Registration [%]	
Rural	38.70
City, rural before	8.93
City	52.37
Grade [%]	
Junior	60.72
Senior	39.28
Ethnicity [%]	
Han	89.36
Others	10.64
Parent Marital Status [%]	
Married	89.04
Separated	0.80
Divorced	6.89
Widowed	2.35
Others	0.91
Parent Highest Education Achievement [%]	
Elementary School and Below	6.90
Junior High School	28.11
High School	25.17
College and above	39.82
Family Income	90,990 (122,030)
Welfare Status	
No	74.72
Yes	25.28
Number of Family Members	3.87 (1.16)
COVID-19 Infection in family and friends [%]	
No	99.14
Infected	0.48
Dead	0.37
Volunteering [%]	74.93
Number of Volunteering	3.29 (4.50)
Hours of Volunteering	24.73 (32.23)
COVID-19 Volunteering [%]	25.12
Number of for COVID-19 Volunteering	0.66 (2.34)
Hours of for COVID-19 Volunteering	4.14 (11.26)
Interplay between public interest and private gains [%]	
High public interest, low private gains	27.74
High public interest and private gains	62.53
Low public interest and high private gains	5.99
Low public interest and private gains	3.74
College [%]	8.05 (4.02)
College 1	7.11
College 2	9.57
College 3	6.25
College 4	10.85
College 5	10.15
College 6	7.06
College 7	6.41
College 8	11.54
College 9	11.12
College 10	2.46
College 11	6.89
College 12	10.58

**Table 2:** Logit Regression Analysis of Volunteering.

	Volunteering						COVID19 Volunteering					
	Model 1			Model 2			Model 3			Model 4		
	OR	S. E.	*p*	OR	S. E.	*p*	OR	S. E.	*p*	B	S. E.	*p*
Interplay between public interest and private gains												
High public interest, low private gains	---	---		5.01	1.42	***	---	---		2.30	0.75	*
High public interest and private gains	---	---		3.45	0.93	***	---	---		1.47	0.47	
Low public interest and high private gains	---	---		2.98	1.03	**	---	---		0.81	0.34	
Low public interest and private gains	---	---		---	---		---	---		---	---	
COVID-19 Infection in family and friends	0.84	0.47		0.78	0.44		2.29	1.22		2.25	1.21	
Female	1.30	0.17	*	1.23	0.16		0.75	0.09	*	0.72	0.09	**
Age	1.13	0.08	+	1.12	0.08		1.22	0.08	**	1.20	0.08	**
Household Registration												
Rural	---	---		---	---		---	---		---	---	
City, rural before	0.81	0.17		0.84	0.18		1.13	0.23		1.19	0.24	
City	0.79	0.13		0.79	0.13		1.04	0.16		1.08	0.17	
Grade: Junior	2.52	0.36	***	2.55	0.37	***	1.49	0.21	***	1.48	0.21	**
Ethnicity: Han	1.31	0.25		1.34	0.26		0.97	0.17		0.98	0.18	
Parent Marital Status												
Married	0.73	0.23		0.73	0.23		1.04	0.29		1.08	0.30	
Divorced	0.73	0.28		0.76	0.29		0.88	0.31		0.93	0.33	
All Other Marital Status	---	---		---	---		---	---		---	---	
Parent Highest Education Achievement												
Elementary School and Below	---	---		---	---		---	---		---	---	
Junior High School	1.45	0.34		1.45	0.35		1.07	0.26		1.07	0.26	
High School	1.49	0.38		1.56	0.40	+	1.55	0.39	+	1.57	0.40	+
College and above	1.27	0.34		1.31	0.36		1.37	0.39		1.40	0.38	
ln (Family Income)	0.97	0.05		0.97	0.06		0.95	0.05		0.97	0.05	
Welfare Status: Yes	1.30	1.19	+	1.27	0.19		1.30	0.18	+	1.28	0.18	+
Number of Family Members	1.00	0.05		1.01	0.06		1.04	0.05		1.05	0.05	
Pseudo R-squared	0.08	□	□	0.10	□	□	0.04	□	□	0.05	□	□

Note: *n* = 1871. + *p* < 0.10; * *p* < 0.05, ** *p* < 0.01, *** *p* < 0.001. College-fixed effects were controlled in all models by including a set of dummy variables for each college in the analysis; □ Pseudo R-squared indicates the proportion of the variance of dependent variable was explained by the covariates; --- indicates the variable did not include in the model.

**Table 3:** OLS Regression of Number of Volunteering.

	ln (Number of Volunteering)			ln (Number of COVID-19 Volunteering)		
	Model 1			Model 2		
	B	S. E.	*p*	B	S. E.	*p*
Interplay between public interest and private gains						
High public interest, low private gains	0.34	0.20	***	0.15	0.16	**
High public interest and private gains	0.27	0.19	***	0.06	0.16	
Low public interest and high private gains	0.11	0.24	**	0.00	0.20	
Low public interest and private gains	---	---		---	---	
COVID-19 Infection in family and friends	−0.01	0.39		0.03	0.32	
Female	0.04	0.08		−0.07	0.07	**
Age	0.02	0.05		0.10	0.04	**
Household Registration						
Rural	---	---		---	---	
City, rural before	−0.02	0.14		0.02	0.11	
Household Registration: City	−0.04	0.10		0.04	0.08	
Grade: Junior	0.17	0.09	***	0.09	0.07	**
Ethnicity: Han	0.02	0.12		0.00	0.10	
Parent Marital Status						
Married	−0.03	0.19		0.00	0.15	
Divorced	−0.02	0.23		−0.02	0.19	
All Other Marital Status	---	---		---	---	
Parent Highest Education Achievement						
Elementary School and Below	---	---		---	---	
Junior High School	0.08	0.16	+	0.02	0.13	
High School	0.09	0.17	*	0.08	0.13	+
College and above	0.07	0.17		0.07	0.14	
ln (Family Income)	0.01	0.04		−0.02	0.03	
Welfare Status	0.05	0.09	*	0.05	0.08	+
Number of Family Members	−0.02	0.03		−0.06	0.03	
Adjusted R-square	0.13	□	□	0.05	□	□

Note: *n* = 1871. + *p* < 0.10; * *p* < 0.05, ** *p* < 0.01, *** *p* < 0.001. College-fixed effects were controlled in all models by including a set of dummy variables for each college in the analysis; □ Pseudo R-squared indicates the proportion of the variance of dependent variable was explained by the covariates; --- indicates the variable did not include in the model.

**Table 4:** OLS Regression of Hours of Volunteering.

	ln (Hours of Volunteering)			ln (Hours of COVID-19 Volunteering)		
	B	S. E.	*p*	B	S. E.	*p*
Interplay between public interest and private gains						
High public interest, low private gains	0.30	0.30	***	0.14	0.26	*
High public interest and private gains	0.27	0.29	***	0.05	0.25	
Low public interest and high private gains	0.11	0.36	**	−0.02	0.31	
Low public interest and private gains	---	---		---	---	
COVID-19 Infection in family and friends	−0.01	0.58		0.02	0.50	
Female	0.04	0.12		−0.07	0.11	**
Age	0.03	0.07		0.07	0.06	*
Household Registration						
Rural	---	---		---	---	
City, rural before	−0.01	0.20		0.01	0.17	
City	−0.05	0.15		0.02	0.13	
Grade: Junior	0.16	0.13	***	0.07	0.12	*
Ethnicity: Han	0.01	0.18		−0.02	0.16	
Parent Marital Status						
Married	−0.04	0.28		0.00	0.24	
Divorced	−0.02	0.34		−0.02	0.29	
All Other Marital Status	---	---		---	---	
Parent Highest Education Achievement						
Elementary School and Below	---	---		---	---	
Junior High School	0.08	0.23	+	0.03	0.20	
High School	0.08	0.25	+	0.10	0.21	*
College and above	0.07	0.26		0.09	0.22	+
ln (Family Income)	0.01	0.05		−0.01	0.05	
Welfare Status	0.04	0.14		0.04	0.12	
Number of Family Members	−0.02	0.05		0.03	0.05	
Adjusted R-square	0.14	□	□	0.05	□	□

Note: *n* = 1871. + *p* < 0.10; * *p* < 0.05, ** *p* < 0.01, *** *p* < 0.001. College fixed effects were controlled in all models by including a set of dummy variables for each college in the analysis; □ Pseudo R-squared indicates the proportion of the variance of dependent variable was explained by the covariates; --- indicates the variable did not include in the model.

## Data Availability

The data presented in this study are available on request from the corresponding author.
